# Dang Gui Bu Xue Tang, a conventional Chinese herb decoction, ameliorates radiation-induced heart disease *via* Nrf2/HMGB1 pathway

**DOI:** 10.3389/fphar.2022.1086206

**Published:** 2023-01-09

**Authors:** Yifan Huang, Minghan Cheng, Xiaoye Wang, Hongliang Dong, Jian Gao

**Affiliations:** ^1^ Pediatric Translational Medicine Institute, Shanghai Children’s Medical Center, School of Medicine, Shanghai Jiao Tong University, Shanghai, China; ^2^ School of Public Health, Suzhou Medical College of Soochow University, Suzhou, China; ^3^ School of Pharmacy, Anhui University of Chinese Medicine, Hefei, China

**Keywords:** myocardial fibrosis (MF), radix angelicae sinensis (RAS), radix astragali membranaceus (RAM), galectin-3 (Gal-3), systolic dysfunction, cardiac injury

## Abstract

**Introduction:** Radiation-induced heart disease (RIHD), characterized by cardiac dysfunction and myocardial fibrosis, is one of the most common complications after cardiothoracic radiotherapy. Dang Gui Bu Xue Tang (DBT) is a conventional Chinese herb decoction composed of Radix Astragali membranaceus (RAM) and Radix Angelicae sinensis (RAS) at a ratio of 5:1, famous for its “blood-nourishing” effect. In this study, we aimed to investigate the cardioprotective effect of DBT on RIHD.

**Methods:** C57BL mice at 8 weeks of age were divided into five groups, namely Control, Radiation, RDBT51 (Radiation with DBT, RAM:RAS = 5:1), RDBT11 (Radiation with DBT, RAM:RAS = 1:1), and RDBT15 (Radiation with DBT, RAM:RAS = 1:5).

**Results:** We mainly found that radiation in the cardiothoracic region led to significant left ventricular systolic dysfunction, myocardial fibrotic lesions and cardiac injury accompanied by abnormally increased myocardial HMGB1 protein levels. Administration of conventional DBT significantly ameliorated left ventricular systolic dysfunction, alleviated myocardial fibrosis, and counteracted cardiac injury, all of which supported the protective effect of DBT on RIHD, involving upregulation of myocardial Nrf2 protein levels and downregulation of HMGB1 protein levels as underlying mechanisms.

**Conclusions:** DBT exerts a significant protective effect on RIHD, and the Nrf2/ HMGB1 pathway probably plays an important role in this protective effect.

## 1 Introduction

Despite being widely used in cancer patients and improving survival rates for many cancers, radiation therapy involves some inevitable complications (Wang et al., 2019; Quintero-Martinez et al., 2021). Radiation-induced myocardial fibrosis, characterized by decreased ventricular elasticity and distensibility, is a potentially lethal clinical complication of chest radiotherapy and a final stage of radiation-induced heart disease (RIHD) (Wang et al., 2020). The development of radiation-induced myocardial fibrosis is a slow but constantly progressive process, with clinical symptoms occurring several years after irradiation. However, the exact underlying mechanism involving radiation-induced myocardial fibrosis is still not completely clarified (Sarkozy et al., 2021), and accurately diagnosing and identifying patients who may progress to radiation-induced myocardial fibrosis has been challenging (Wang et al., 2020).

High mobility group box 1 (HMGB1), a highly conservative nucleoprotein, was a multifunctional protein involved in the regulation of inflammation, cancer and fibrosis progression (Wang and Zhang, 2020). As an important late inflammatory cytokine, the role of HMGB1 in fibrotic diseases has become one of the hot issues during the past years. The expression of HMGB1 is up-regulated in pulmonary fibrosis and participates in the occurrence and development of pulmonary fibrosis. Many studies have shown that inhibiting the production of HMGB1 can effectively reduce the inflammatory reaction-related damage in the body. Our previous studies demonstrated that Nrf2 attenuated the epithelial mesenchymal transition process in pulmonary fibrosis through inhibition of HMGB1 pathway (Zhang et al., 2018; Qu et al., 2019).

The role of HMGB1 in cardiovascular disease and cardiac fibrosis has been increasingly concerned in recent years, and a series of progress has also been made. Many studies have shown that HMGB1 acts on the RAGE receptor on the surface of cardiac fibroblasts, making it form heterodimer with some TLRs (such as TLR2/4/9) or β - integrin Mac-1, and stimulate cardiac fibroblasts to obtain the characteristics of proliferation, migration and fibrosis promotion through NF-κB, MAPKs and JAK/STAT (Turner, 2016). In the cardiac fibrosis model induced by diabetes, silencing HMGB1 can inhibit cardiac fibrosis and improve cardiac function through MAPK pathway (Wang et al., 2014). All these data showed that HMGB1 is a very promising target for cardiac fibrosis treatment, but whether the therapeutic strategy targeting HMGB1 plays a protective role in patients still needs more evidence. In RIHD, whether HMGB1 is involved in the process of myocardial injury and cardiac fibrosis is still unknown.

Dang Gui Bu Xue Tang (DBT), a combination of *Radix Astragali membranaceus* (RAM) and *Radix Angelicae sinensis* (RAS) at a conventional ratio of 5:1, is a widely used herbal decoction in traditional Chinese medicine promote or invigorate the “blood”. DBT has been proved to mediate potent cardioprotective and anti-cardiac fibrosis effects (Mak et al., 2006), while the underlying mechanisms remain to be elaborated. In the present study, we developed *in vivo* and *in vitro* models of RIHD. Through these models, we revealed that DBT-mediated Nrf2 activation could downregulate radiation-induced increase in cardiac HMGB1, improve abnormal weight loss, and reduce cardiac fibrosis. These findings uncovered a Nrf2-HMGB1 axis mediating the protective effect of DBT on cardiac fibrosis, providing a useful clue for a clinical strategy against the disease.

## 2 Materials and methods

### 2.1 Animal and cell preparation

The animal study was reviewed and approved by the Animal Ethics Committee of Soochow University. A total of 45 male C57BL mice at 7 weeks of age were purchased from the Beijing Vital River Laboratory (Beijing Vital River Laboratory Animal Technology Co., Ltd., China). All mice were housed in a temperature- and humidity-controlled facility without specific pathogen at 12-h light and dark cycles, with water and food *ad libitum*.

Neonatal mice (born within 48 h, Beijing Vital River Laboratory Animal Technology Co., Ltd., China) were euthanized by decapitation and then the neonatal mouse myocardial fibroblasts were isolated and cultured according to the methods we described previously (Zhang et al., 2022). Briefly, mouse ventricular myocardial tissues were minced and rinsed in D-Hanks balanced salt solution, and subsequently digested in D-Hanks solution containing 10 μg/ml Liberase TH (Roche, 5,401,151,001), incubated at 37°C for 30 min. High glucose Dulbecco’s Modified Eagle’s Medium (DMEM) (HyClone, United States of America) supplemented with 10% foetal bovine serum (FBS) (Gibco, United States of America) was added to the digested supernatant to terminate the digestion, followed by centrifugation at 100 *g* for 10 min to collect suspended cells. All isolated cells were pre-plated in a humidified incubator at 37°C with 5%CO_2_ for 1 hour, after which the unattached cells were discarded and the attached cells were myocardial fibroblasts. Isolated myocardial fibroblasts were cultured in high glucose DMEM with 10% FBS for further *in vitro* experiments.

### 2.2 DBT preparation

The preparation of DBT was based on previous studies (Chunhua et al., 2017; Zhou et al., 2018). In short, a total weight of 600 g RAM and RAS at ratios of 5:1 (DBT51), 1:1 (DBT11), and 1:5 (DBT15) were immersed respectively for 1 hour with 8 times the volume of distilled water (4.8 L). The medicinal materials were decocted twice at 100°C for 1 hour, and subsequently the decocted liquids were collected and merged together. After filtration, the decocted liquid was concentrated to 500 ml by water bath, resulting in a final DBT concentration of 1.2 g/ml. For cell experiments, some DBT was diluted to a final concentration of 1 mg/ml by using DMEM with 2% FBS and sterilized by filtration (Zhou et al., 2018).

### 2.3 RIHD model and DBT treatment

#### 2.3.1 In vivo study

After 1 week adaptation period, all mice were randomly divided into five groups: Control (*n* = 5); Radiation (*n* = 10); RDBT51 (Radiation with DBT, RAM:RAS = 5:1, *n* =10); RDBT11 (Radiation with DBT, RAM:RAS = 1:1, *n* = 10); RDBT15 (Radiation with DBT, RAM:RAS = 1:5, *n* = 10). The precordial area of each mouse in radiation groups was exposed to X-ray irradiation individually to establish a murine RIHD model. After all mice had been anesthetized with isoflurane anaesthesia (2%) using a mask, the hair on the chest was removed, and irradiation was applied with a 6 MV X-ray beam energy at a single dose of 20 Gy using an X-RAD 320ix Irradiator (Precision X-Ray Inc., United States). For 8 consecutive weeks starting from the day of radiation, distilled water or diverse DBT (10 μl/g body weight, equivalent to 12 g/kg body weight) were administered to mice once daily *via* oral gavage. This DBT dose of 12 g/kg was designed based on the previous study (Chunhua et al., 2017; Liu et al., 2018) and our preliminary experiments. All mice were sacrificed at the end of the 8 weeks after irradiation. Mouse serum was obtained by centrifugation of blood at 1000× g for 10 min at 4°C, and cardiac tissue was isolated and stored for the following experiments.

#### 2.3.2 In vitro study

Primary neonatal mouse myocardial fibroblasts cultured in high glucose DMEM with 10% FBS were seeded in 6-well plate and randomly divided into five groups: Control; Radiation; RDBT51 (Radiation with DBT, RAM:RAS = 5:1); RDBT11 (Radiation with DBT, RAM:RAS = 1:1); RDBT15 (Radiation with DBT, RAM:RAS = 1:5). At 70%–80% confluency, myocardial fibroblasts in radiation groups were irradiated with a single dose of 4 Gy at a dose rate of 2 Gy/min using an X-RAD 320ix Irradiator (Precision X-Ray Inc., United States). After administration, myocardial fibroblasts immediately received fresh control culture medium or concerning DBT medium (1 mg/ml) with 2%FBS. The specific inhibitor ML385 (1 μM, MCE, United States of America) was used to inhibit Nrf2 in fibroblasts. After 24 or 48 h, culture medium was collected and centrifuged at 10,000 g for 5 min, and then the supernatant was kept for further ELISA assay.

### 2.4 Echocardiography

At 4 and 8 weeks after irradiation, the mice were anesthetized with inhaled isoflurane (1%) and imaged using a 40 MHz linear array transducer attached to a preclinical ultrasound system (Vevo 2100, Fujifilm VisualSonics, Toronto, ON, Canada) with nominal in-plane spatial resolution of 40 µm (axial) × 80 µm (lateral). M-mode and 2-D parasternal short-axis scans (133 frames/second) at the level of the papillary muscles were employed to assess the changes in left ventricular (LV) end-systolic inner diameter, LV end-diastolic inner diameter, LV posterior wall thickness in end-diastole and end-systole, fractional shortening (FS), and ejection fraction (EF).

### 2.5 Histological analysis

Cardiac tissues were routinely collected, fixed, processed and sectioned into 5 µm slices. The cardiac collagen deposition was determined by Sirius red (Shanghai Yuanye Biotechnology, Shanghai, China) staining as previously described (Zhang et al., 2022).

### 2.6 Western blot and real-time qPCR analysis

Cardiac tissues or myocardial fibroblasts were harvested in RIPA Digest Buffer. The supernatant was collected, and protein concentration was measured using the BCA Protein Assay Reagent Kit. Equal amounts of protein were electrophoretically separated in SDS-polyacrylamide gels and then transferred onto polyvinylidene difluoride membranes (PVDF, IPVH00010, Millipore, United States). The blot was blocked with 5% skim milk for 1 h at room temperature and probed overnight at 4°C by incubation with the primary antibodies, including anti-Nrf2 (ab31163, Abcam, Cambridge, United Kingdom), anti-HMGB1 (3935, CST, Boston, United States of America) and anti-GAPDH (2118, CST, Boston, United States of America) antibodies. After being washed with Tris Buffered Saline with Tween (TBST), the membranes were subsequently incubated with secondary horseradish peroxidase conjugated anti-rabbit antibodies at 25°C for 2 h. Finally, membranes were analysed. Total RNA was extracted using the TRIzol reagent (Takara, Tokyo, Japan) according to the manufacturer’s instructions, and then was analysed *via* the real-time qPCR as described before (Zhang et al., 2022). The sequences of primers are as follows: *Collagen-I,* Forward-GCAAGAGGCGAGAGAGGTTT and Reverse-TGCACCACCAACTGCTTAGC; *Collagen-III,* Forward-ACGTAGATGAATTGGGATGCAG and Reverse-GGGTTGGGGCAGTCTAG; *TGF-β,* Forward-TGGGCACCATCCATGACAT and Reverse-TCTTCTCTGTGGAGCTGAAGCA; *GAPDH,* Forward-GGATGCAGGGATGATGTTCT and Reverse-TGCACCACCAACTGCTTAGC.

### 2.7 ELISA

The serum levels of HMGB1 and Galectin-3 were assayed by using ELISA kits (eBioscience, United States) according to the manufacturer’s instructions. Standard curves were established using mouse recombinant cytokines provided in the kits.

### 2.8 Statistical analysis

Data are presented as mean ± standard deviation. The analysis of variance followed by the Bonferroni test was adopted for multiple comparisons among groups.

## 3 Results

### 3.1 The DBT protected mice against radiation-induced weight loss

**FIGURE 1:**
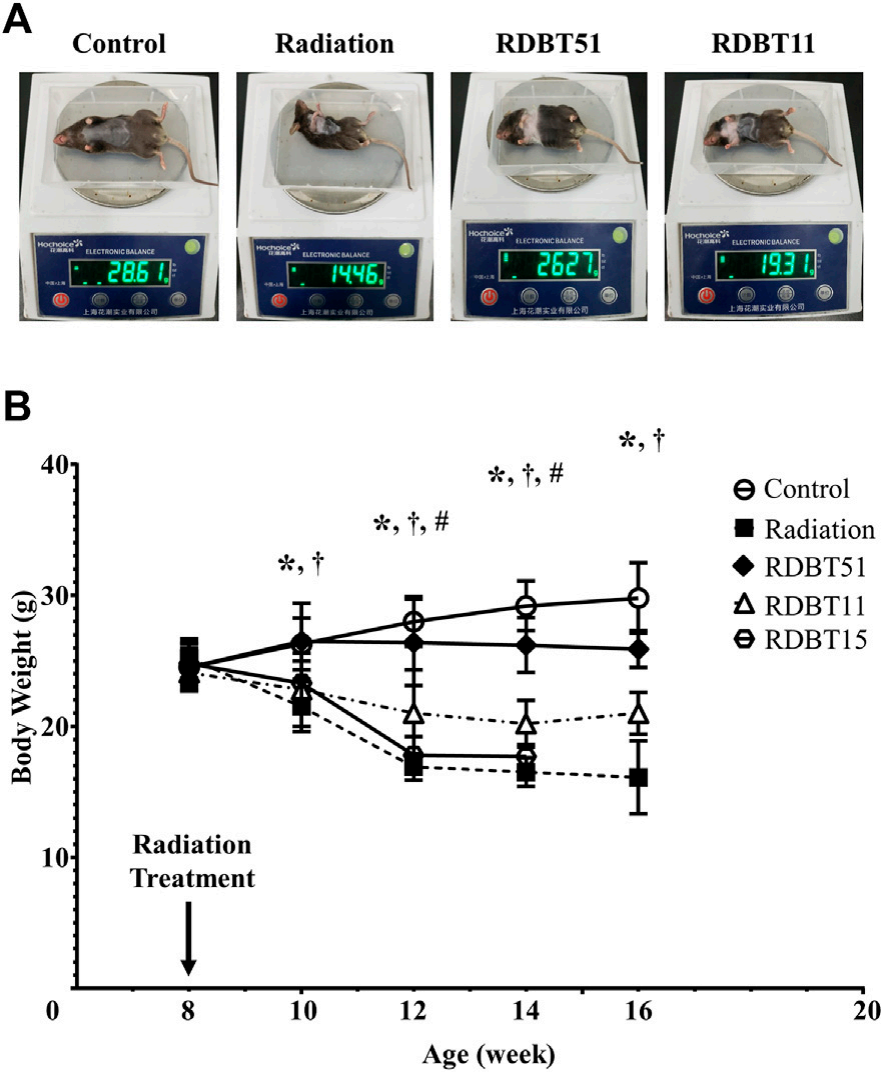
The protective effect of DBT on the radiation-induced abnormal weight loss. **(A)** The representative images of body weighing at 8 weeks after radiation; **(B)** Trends in body weight change. *p < 0.05 of Radiation versus Control; ^†^ *p* < 0.05 of RDBT51 versus Radiation; ^#^ *p* < 0.05 of RDBT11 versus Radiation. DBT, Dang Gui Bu Xue Tang; RAM, *Radix Astragali membranaceus*; RAS, *Radix Angelicae sinensis*; RDBT51, Radiation with DBT (RAM: RAS = 5:1); RDBT11, Radiation with DBT (RAM: RAS = 1:1); RDBT15, Radiation with DBT (RAM: RAS = 1:5); The “R” in the “RDBT” stands for Radiation, while the two digits following RDBT represent the ratio between RAM and RAS, the same below.

The radiation treatment led to an obvious body weight loss in mice, as shown in Figure 1. The body weight of mice in the radiation group started to be significantly lower than that in the control group from 2 weeks after radiation and continued through 8 weeks (all *p* < 0.05). The body weight of mice in the RDBT51 group was consistently significantly higher than that of mice in the radiation group (all *p* < 0.05). Similarly, the mice in the RDBT11 group inclined to have higher body weights than those in the radiation group, but only at 4 and 6 weeks after radiation there were significant differences in body weight between the radiation and RDBT11 group.

### 3.2 The DBT reduced radiation-induced elevation of serum inflammatory and myocardial injury biomarkers

**FIGURE 2:**
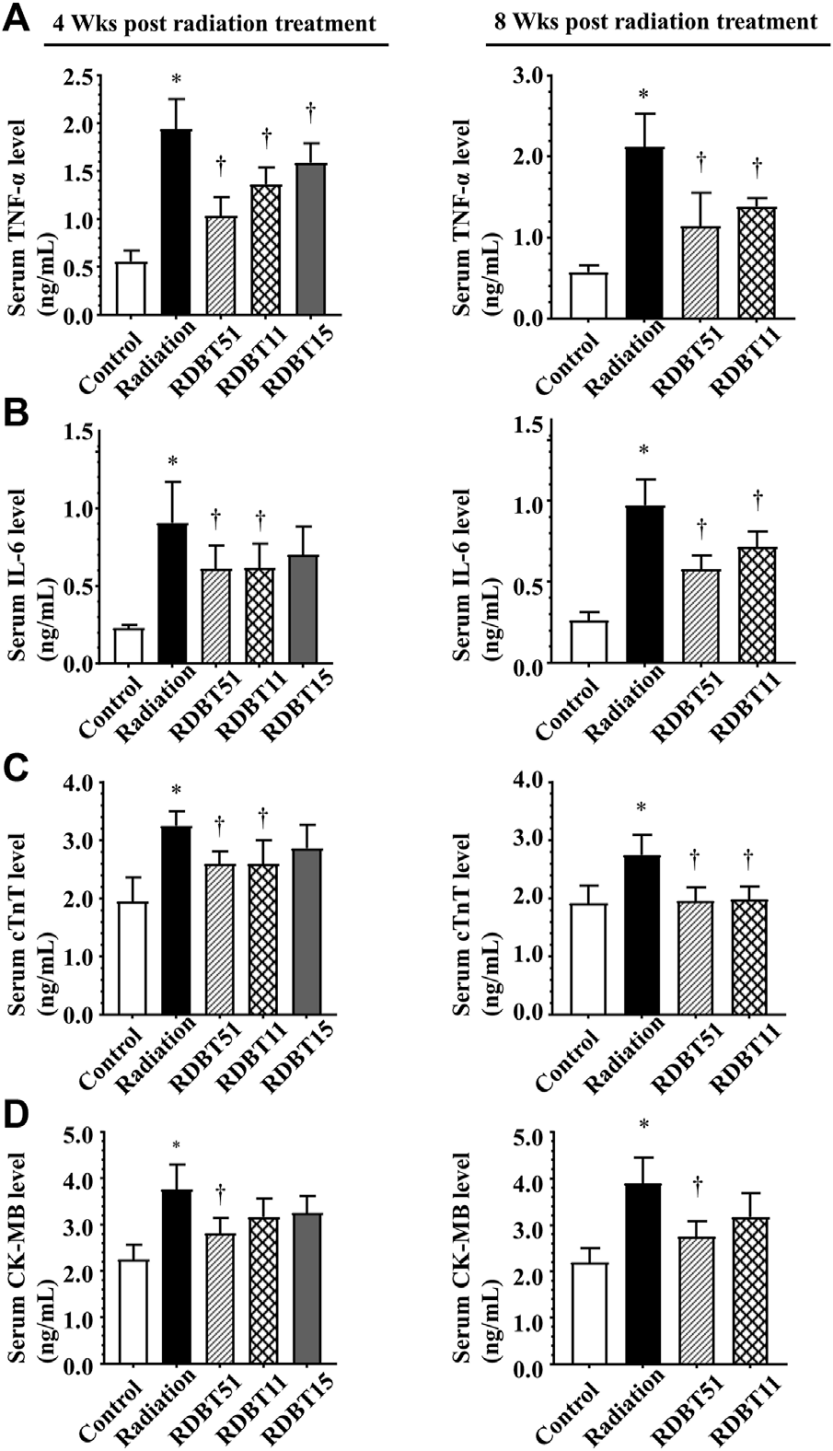
The DBT ameliorates the inflammation- and myocardial injury-related biomarkers in the serum of mice at 4 and 8 weeks after radiation. **(A)** TNF-α; **(B)** IL-6; **(C)** cTnT; **(D)** CK-MB. *p < 0.05 versus Control; ^†^ *p* < 0.05 versus Radiation. DBT, Dang Gui Bu Xue Tang; RAM, *Radix Astragali membranaceus*; RAS, *Radix Angelicae sinensis*; RDBT51, Radiation with DBT (RAM: RAS = 5:1); RDBT11, Radiation with DBT (RAM: RAS = 1:1); RDBT15, Radiation with DBT (RAM: RAS = 1:5).

As shown in Figure 2, the radiation treatment led to a significant elevation of serum inflammatory biomarkers, *i.e.,* TNF-α and IL-6, as well as myocardial injury biomarkers such as cTnT and creatine kinase - MB (CK-MB), at 4 and 8 weeks after radiation (all *p* < 0.05). Notably, the serum levels of TNF-α, IL-6, cTnT, and CK-MB were consistently lower in the RDBT51 group than in the radiation group (all *p* < 0.05). Except for CK-MB, the above biomarkers were also lower in the RDBT11 group than in the radiation group, while there was no significant difference in the serum IL-6, cTnT, and CK-MB levels between the mice in the radiation and RDBT15 group.

### 3.3 The DBT improved radiation-induced myocardial systolic dysfunction

**FIGURE 3:**
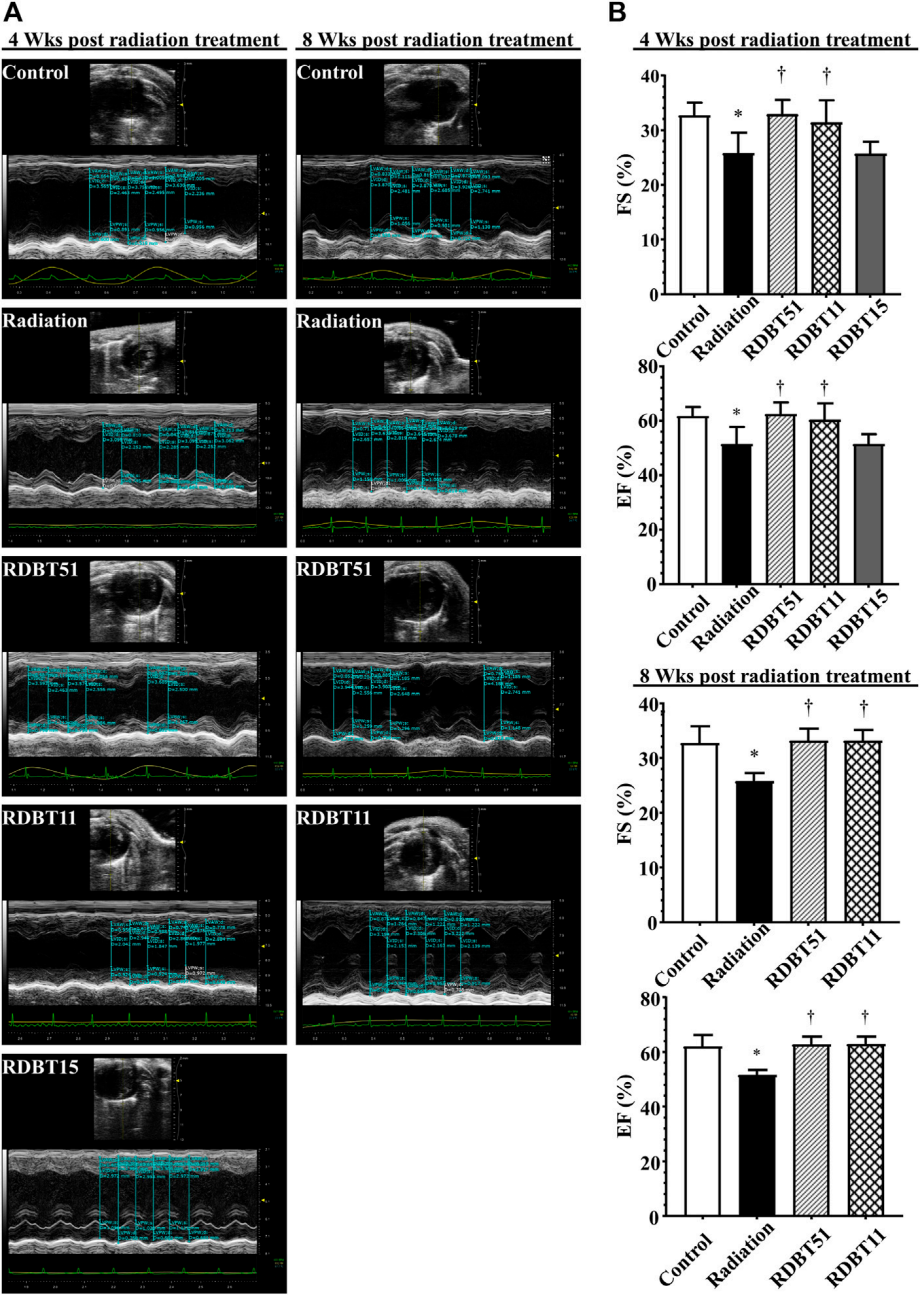
The DBT improves radiation-induced left ventricular systolic dysfunction. **(A)** Representative echocardiograms obtained at 4 and 8 weeks after radiation treatment; **(B)** Quantitative statistical analyses of FS and EF. The other concerning quantitative statistical analyses of cardiac function related parameters are summarized in Table 1. DBT, Dang Gui Bu Xue Tang; EF, ejection fraction; FS, fractional shortening; RAM, *Radix Astragali membranaceus*; RAS, *Radix Angelicae sinensis*; RDBT51, Radiation with DBT (RAM: RAS = 5:1); RDBT11, Radiation with DBT (RAM: RAS = 1:1); RDBT15, Radiation with DBT (RAM: RAS = 1:5).

The improvement effect of DBT on radiation-induced left ventricular systolic dysfunction was summarized in Figure 3 and Table 1. The mice in the radiation group had lower EF and FS compared with the mice in the control group, accordingly, the EF and FS of mice in both RDBT51 and RDBT11 groups were significantly higher than those in the radiation group (all *p* < 0.05). Nevertheless, significant differences were found neither in EF nor in FS between the radiation and RDBT15 group.

**TABLE 1:** Left ventricular systolic function and morphologic data obtained from echocardiographic measurement.

	4 weeks					8 weeks			
	Control	Radiation	RDBT51	RDBT11	RDBT15	Control	Radiation	RDBT51	RDBT11
LVAWd (mm)	0.68 ± 0.08	0.73 ± 0.09	0.74 ± 0.17	0.69 ± 0.07	0.58 ± 0.27	0.81 ± 0.06	0.63 ± 0.12	0.66 ± 0.15	0.72 ± 0.17
LVAWs (mm)	1.02 ± 0.12	0.95 ± 0.15	1.04 ± 0.20	0.94 ± 0.05	0.90 ± 0.26	1.08 ± 0.10	0.87 ± 0.21	0.93 ± 0.23	1.01 ± 0.21
LVIDd (mm)	3.82 ± 0.26	3.66 ± 0.46	3.35 ± 0.48	3.32 ± 0.38	3.55 ± 0.46	3.79 ± 0.18	3.63 ± 0.52	3.67 ± 0.32	3.47 ± 0.25
LVIDs (mm)	2.57 ± 0.16	2.72 ± 0.40	2.26 ± 0.39	2.28 ± 0.29	2.64 ± 0.36	2.55 ± 0.14	2.69 ± 0.33	2.45 ± 0.17	2.31 ± 0.14
LVPWd (mm)	0.66 ± 0.08	0.67 ± 0.05	0.65 ± 0.15	0.64 ± 0.07	0.68 ± 0.09	0.72 ± 0.14	0.66 ± 0.10	0.68 ± 0.19	0.67 ± 0.12
LVPWs (mm)	1.04 ± 0.14	0.94 ± 0.15	0.94 ± 0.12	0.92 ± 0.08	1.01 ± 0.08	1.07 ± 0.11	0.92 ± 0.15	1.10 ± 0.33	0.97 ± 0.04
IVSd (mm)	0.69 ± 0.08	0.74 ± 0.09	0.74 ± 0.17	0.70 ± 0.07	0.58 ± 0.27	0.81 ± 0.06	0.63 ± 0.12	0.67 ± 0.15	0.73 ± 0.17
IVSs (mm)	1.02 ± 0.09	1.00 ± 0.09	1.11 ± 0.21	1.03 ± 0.06	0.73 ± 0.38	1.20 ± 0.15	0.88 ± 0.23	0.96 ± 0.29	1.11 ± 0.29

Data are presented as mean ± standard deviation, N = 5–7/group. Abbreviations: DBT, Dang Gui Bu Xue Tang; RAM, *Radix Astragali membranaceus*; RAS, *Radix Angelicae sinensis*; RDBT51, Radiation with DBT (RAM: RAS, 5:1); RDBT11, Radiation with DBT (RAM: RAS, 1:1); RDBT15, Radiation with DBT (RAM: RAS, 1:5); LVAW, left ventricular anterior wall; LVID, left ventricular internal diameter; LVPW, left ventricular posterior wall; IVS, interventricular septum; -d, in diastole; -s, in systole.

### 3.4 The DBT alleviated radiation-induced myocardial fibrosis

**FIGURE 4:**
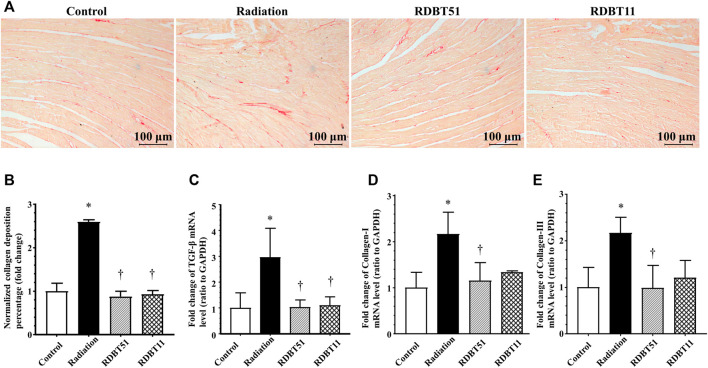
The DBT alleviates radiation-induced cardiac fibrosis. **(A)** Representative microphotographs of Sirius red staining obtained at 8 weeks after radiation; **(B)** Quantitative results of collagen deposition percentage; **(C)**, **(D)**, and **(E)** The myocardial mRNA levels of TGF-β, Collagen-I and Collagen-III relative to GAPDH were analysed by real-time qPCR. *p < 0.05 versus Control; ^†^ *p* < 0.05 versus Radiation. DBT, Dang Gui Bu Xue Tang; RAM, *Radix Astragali membranaceus*; RAS, *Radix Angelicae sinensis*; RDBT51, Radiation with DBT (RAM: RAS = 5:1); RDBT11, Radiation with DBT (RAM: RAS = 1:1).

The beneficial effect of DBT on radiation-induced myocardial fibrosis were summarized in Figure 4, which were comparable to the results concerning myocardial systolic function. The mice in the radiation group had a significantly higher degree of myocardial fibrosis compared with the mice in the control group, based on the myocardial collagen deposition and the mRNA levels of TGF-β, Collagen-I, and Collagen-III across groups (all *p* < 0.05). On this basis, the degree of myocardial fibrosis was lower in the RDBT51 group than in the radiation group. Meanwhile, the mice in the RDBT11 group also had a lower degree of myocardial fibrosis compared with the mice in the control group, despite the fact that there was no significant difference between groups in the mRNA level of myocardial Collagen-I and Collagen-III.

**FIGURE 5:**
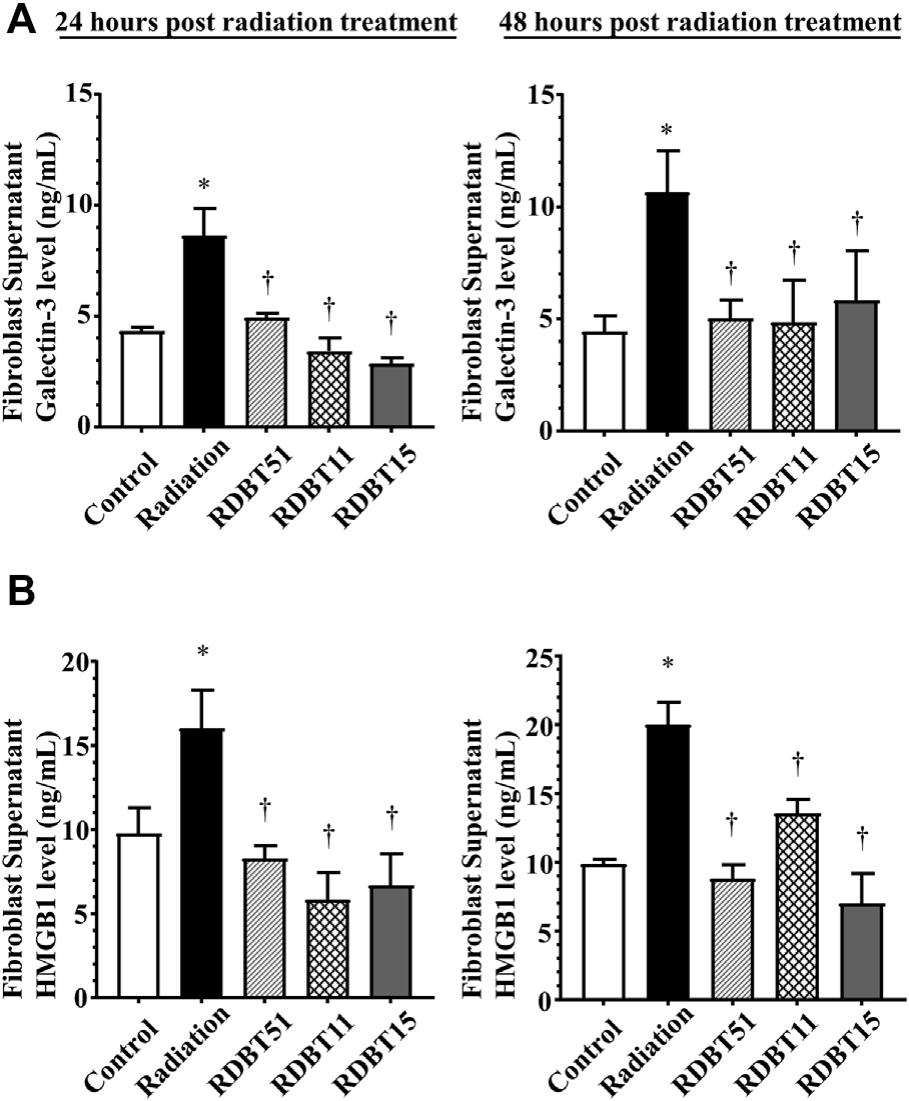
The DBT reduces the fibrosis-related biomarkers in the neonatal mouse myocardial fibroblast culture supernatant at 24 and 48 h after radiation. **(A)** Galectin-3; **(B)** HMGB1. *p < 0.05 versus Control; ^†^ *p* < 0.05 versus Radiation. DBT, Dang Gui Bu Xue Tang; HMGB1, high mobility group box 1; RAM, *Radix Astragali membranaceus*; RAS, *Radix Angelicae sinensis*; RDBT51, Radiation with DBT (RAM: RAS = 5:1); RDBT11, Radiation with DBT (RAM: RAS = 1:1); RDBT15, Radiation with DBT (RAM: RAS = 1:5).

**FIGURE 6:**
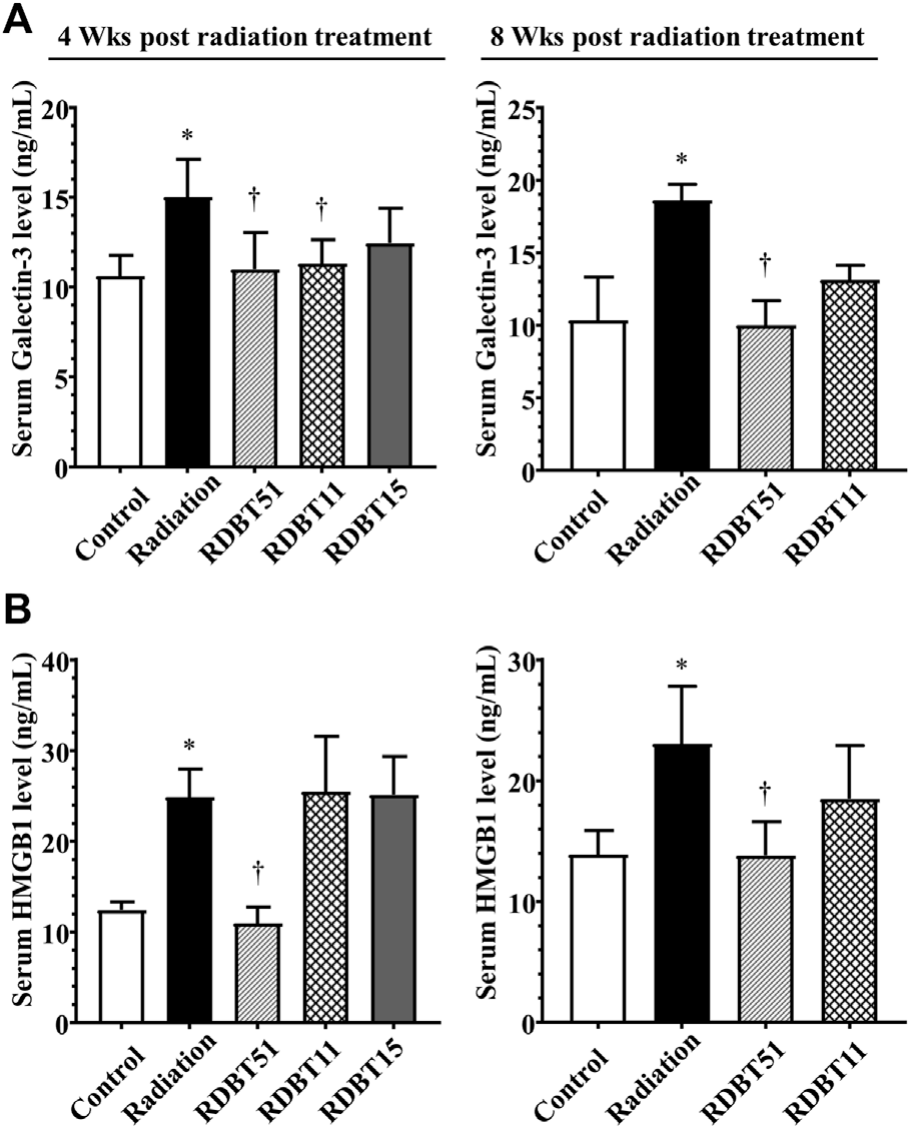
The DBT reduces the fibrosis-related biomarkers in the serum of mice at 4 and 8 weeks after radiation. **(A)** Galectin-3; **(B)** HMGB1. *p < 0.05 versus Control; ^†^ *p* < 0.05 versus Radiation. DBT, Dang Gui Bu Xue Tang; HMGB1, high mobility group box 1; RAM, *Radix Astragali membranaceus*; RAS, *Radix Angelicae sinensis*; RDBT51, Radiation with DBT (RAM: RAS = 5:1); RDBT11, Radiation with DBT (RAM: RAS = 1:1); RDBT15, Radiation with DBT (RAM: RAS = 1:5).

The results derived from assessment of myocardial fibroblasts supernatant and serum Galectin-3 levels, the indicator of fibrosis, were consistent with the differences in myocardial fibrosis among groups described above (Figure 5 and Figure 6). At 24 and 48 h after radiation treatment, the levels of Galectin-3 were significantly higher in the fibroblast culture medium of the radiation group than that in the fibroblast culture medium of the control, RDBT51, and RDBT11 groups (Figure 5, all *p* < 0.05). In parallel, at 4 weeks and 8 weeks after radiation treatment, the mouse serum levels of Galectin-3 were also significantly higher than those in the control and RDBT51 groups (Figure 6, all *p* < 0.05). The above differences were neither observed between the radiation and RDBT11 group, nor between the radiation and RDBT15 group, except for the serum Galectin-3 level between the radiation and the RDBT11 group at the time point of 4 weeks post-radiation.

### 3.5 The DBT increased myocardial Nrf2 expression and decreased the radiation-induced elevated expression of myocardial HMGB1

**FIGURE 7:**
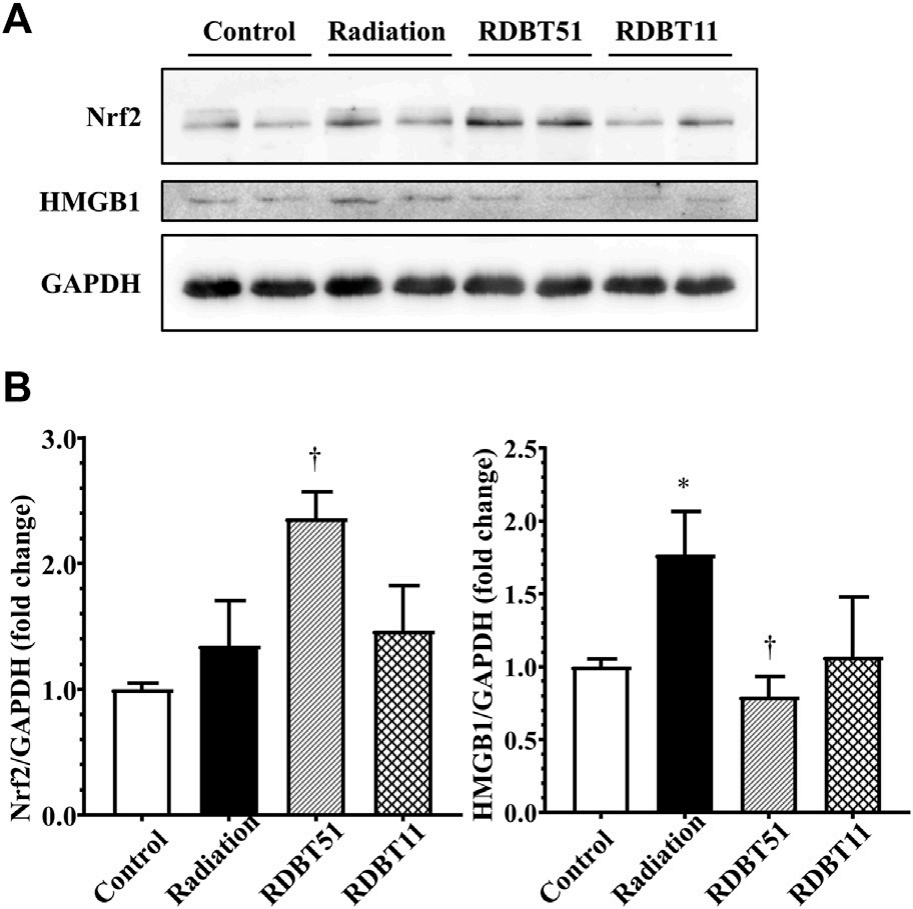
The regulatory effect of radiation and DBT on mouse myocardial Nrf2 and HMGB1 protein levels. **(A)** Representative western blot bands; **(B)** Quantitative results of Western blot. *p < 0.05 versus Control; ^†^ *p* < 0.05 versus Radiation. DBT, Dang Gui Bu Xue Tang; HMGB1, high mobility group box 1; RAM, *Radix Astragali membranaceus*; RAS, *Radix Angelicae sinensis*; RDBT51, Radiation with DBT (RAM: RAS = 5:1); RDBT11, Radiation with DBT (RAM: RAS = 1:1).

As shown in Figure 5 and Figure 6, the levels of HMGB1 in fibroblast culture supernatant or mouse serum of the radiation group were highest among fibroblast culture groups or mouse groups at the corresponding time points. Correspondingly, at the end of 8 weeks after radiation treatment, the myocardial HMGB1 protein level in the radiation group, as well as the myocardial Nrf2 protein level in the RDBT51 group, was highest among all mouse groups. Although the myocardial HMGB1 protein level in the RDBT11 group tended to be lower than that in the radiation group, the difference was not significant (Figure 7).

**FIGURE 8:**
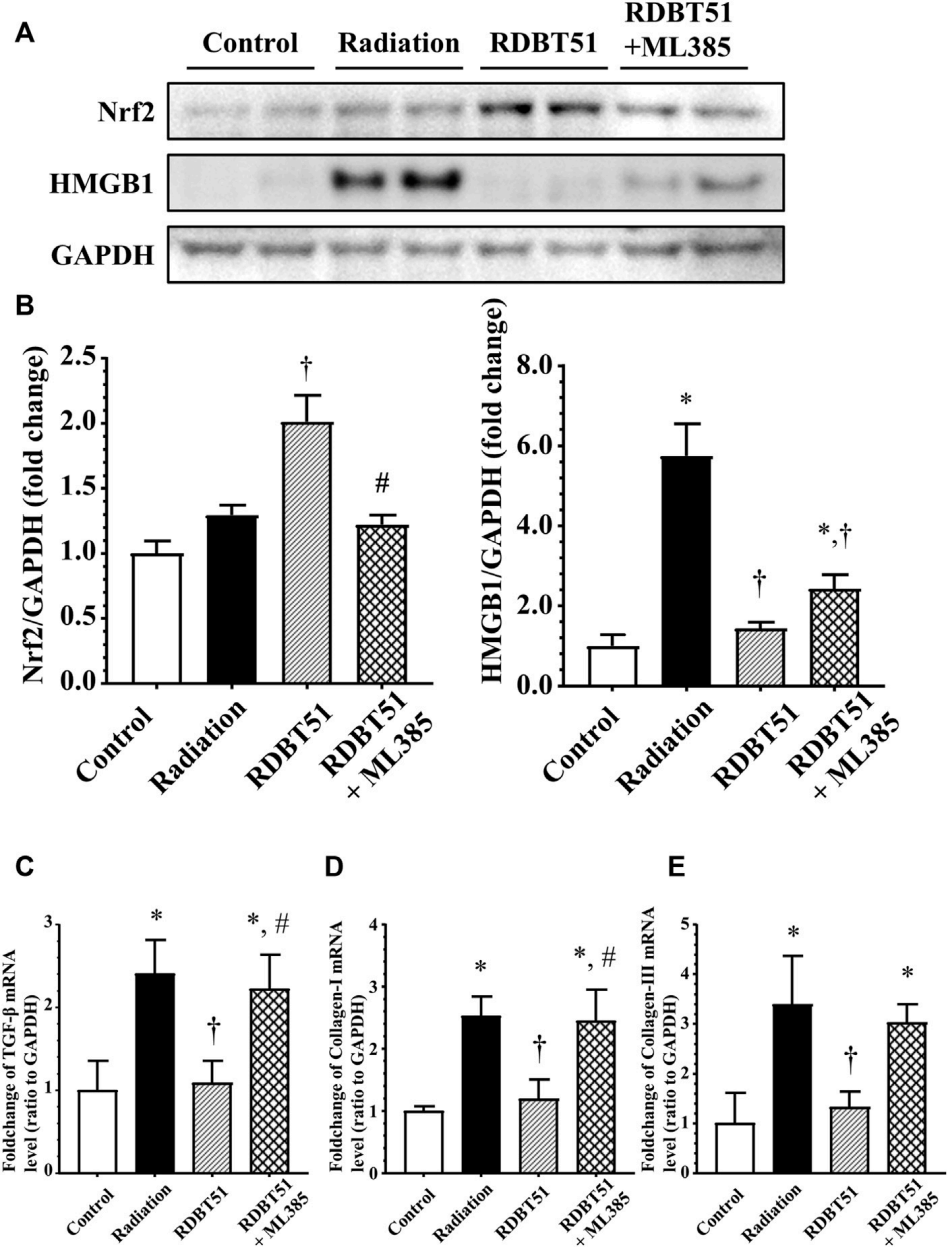
Inhibition of Nrf2 compromises the protective effect of DBT on radiation-induced deleterious changes in myocardial fibroblasts. After administration of radiation, myocardial fibroblasts were immediately changed to fresh normal medium, DBT51 medium (1 mg/ml), or DBT51 medium with 1 μM ML385 (specific inhibitor of Nrf2) for 48 h and then collected for analyses. **(A)** Representative western blot bands; **(B)** Quantitative results of Western blot; **(C)**, **(D)**, and **(E)** Quantitative results of real-time qPCR for TGF-β, Collagen-I and Collagen-III. *p < 0.05 versus Control; ^†^ *p* < 0.05 versus Radiation; ^#^ *p* < 0.05 versus DBT51. DBT, Dang Gui Bu Xue Tang; HMGB1, high mobility group box 1; RAM, *Radix Astragali membranaceus*; RAS, *Radix Angelicae sinensis*; RDBT51, Radiation with DBT (RAM: RAS = 5:1).

By inhibiting Nrf2 *via* ML385 in fibroblasts in the RDBT group, we furthermore validated the mediating role of Nrf2/HMGB1 in the protective effect of DBT. As shown in Figure 8, in line with the results from the *in vivo* study, the level of Nrf2 was higher in the RDBT51 group than in the radiation and RDBT51 + ML385 groups, whereas the expression levels of HMGB1, TGF-β, Collagen-I and Collagen-III were higher in the RDBT51 + ML385 group than in the RDBT group (all *p* < 0.05).

## 4 Discussion

In this study, we mainly found the significant deleterious side effect of radiation treatment in cardiothoracic region, which is characterized by body weight loss, cardiac systolic dysfunction and myocardial fibrosis, can be effectively ameliorated by the supplementation of DBT. In addition, the implicated abnormal expression of Nrf2, HMGB1 and Galectin-3 may play important roles during the whole disease development.

### 4.1 Exploration of the rationality of RAM to RAS ratio

Previous studies have demonstrated that the application of RAM and RAS individually, or in combination, may exert potential cardioprotective effect through anti-inflammatory and anti-oxidative mechanisms. In this study, we designated three herb weight-to-weight ratios, namely RAM and RAS in the ratios of 5:1 (conventional formulation of DBT), 1:1 and 1:5, in order to investigate whether RAM and RAS protect against RIHD, furthermore, to compare the efficacy differences across ratios. The results revealed that the DBT with a ratio of RAM to RAS at 5:1 most effectively ameliorated the abnormal weight loss, myocardial injury and fibrosis caused by radiation treatment among three ratio groups, supporting the rationality of the conventional formulation.

Numerous previous studies also demonstrated the rationality of maintaining the RAM to RAS ratio at 5:1. Dong *et al.* found that the contents of main active components such as astragaloside IV, calycosin, formononetin and ferulic acid were highest when the extraction ratio of RAM to RAS was 5:1, and the DBT extracted in this ratio achieved the best chemical composition and biological effect (Dong et al., 2006). In parallel, Mak *et al.* found that the RAM extracts seemed to play more important roles than RAS extracts in DBT, since RAM extracts contained more bioactive components than RAS extract (Mak et al., 2006). Thus, it is understandable that Liu *et al.* found that the DBT based on this ratio exerted a significant ameliorative effect on heart injury, including inhibition of the NF-κB pathway as probable mechanisms (Liu et al., 2018). To a certain degree, the above studies also provide the probable reasons why conventional DBT (RAM: RAS = 5:1) has the most significant beneficial effect among groups.

### 4.2 The Nrf2/HMGB1 pathway in the development of RIHD

During the past years, emerging evidence indicates that increased HMGB1 is closely associated with fibrotic disease models, and moreover, targeted inhibition of HMGB1-related pathways significantly resists fibrosis. For instance, Wu *et al.* found that inhibition of HMGB1 significantly counteracted the isoproterenol-induced cardiac fibrosis by recovering TLR2-mediated autophagy suppression (Wu et al., 2018). Likewise, we demonstrated in the rat model of pulmonary fibrosis, the increased expression of HMGB1 was closely associated with the generation of extracellular matrix components, accompanied by increased expression of TGF-β (Li et al., 2015). Nrf2 has been considered to be a robust therapeutic target for cardiovascular diseases because of its ability to regulate the expression of numerous antioxidants. It is noteworthy that our previous results suggested the regulatory role of Nrf2 for HMGB1. Based on Nrf2 knockout mice, we verified that Nrf2 activation remarkably downregulated the expression of HMGB1, inhibited the TGF-β-induced epithelial-mesenchymal transition and reactive oxygen species generation, subsequently ameliorated the development of pulmonary fibrosis (Qu et al., 2019). Considering that in the present study, myocardial Nrf2 levels were lower, while HMGB1 and TGF-β levels were higher in the radiation group than that in the control group, it is reasonable to infer the Nrf2/HMGB1 pathway at least partially contributes to the radiation-induced myocardial pathological changes.

### 4.3 The underlying mechanism of DBT resistance to RIHD

According to previous relevant studies, DBT contains sorts of biological active ingredients including astragaloside IV, calycosin, formononetin, ferulic acid, total flavonoids, total saponins, and total polysaccharides (Dong et al., 2006). Astragaloside IV has been demonstrated to attenuate the inflammatory reaction *via* inhibition of HMGB1 (Huang et al., 2012; Li et al., 2016). Decreased HMGB1 level was observed in livers from mice fed with calycosin enhanced diet (Cheng et al., 2020), while in a previous *in vitro* study, administration of formononetin also led to downregulation of HMGB1 (Wang et al., 2018). When it comes to total flavonoids, Shen *et al.* have investigated the interaction between quercetin, isoquercitrin, rutin and HMGB1 through a comprehensive spectral and *in silico* analysis, and eventually they concluded that all the above three flavonoids could directly interact with HMGB1, caused conformational changes and then reduced pro-inflammatory activity of HMGB1.

Couples of studies also have investigated the effect of DBT, or some of its main active ingredients, on Nrf2. Results from Li *et al.* showed that RAM-derived astragaloside IV activated Nrf2 signaling pathway and attenuated inflammatory response (Li H et al., 2018). Li *et al.* described that calycosin and ferulic acid probably contribute to the protective of Yiqi Huoxue Decoction on ischemia/hypoxia-induced oxidative stress injury in H9c2 cardiomyocytes, involving increased Nrf2 expression as underlying mechanisms (Li F et al., 2018). Additionally, flavonoids were reported to be capable of activating Nrf2 pathway in many disease models (Suraweera et al., 2020). In terms of polysaccharides, results of several studies similarly suggested the regulatory function of astragalus polysaccharides on Nrf2 (Farag et al., 2019; Zhang et al., 2021).

However, it is important to note that, to date, few studies have directly investigated the cardioprotective effect of DBT under the radiation condition. Furthermore, many data on the regulatory role of DBT in Nrf2/HMGB1 pathway have come from other organs, with very limited data from the heart. Our results suggested that DBT may be a promising clinical treatment for RIHD, but more population studies, especially well-designed randomized double-blind controlled trials, are needed to verify it in future.

In conclusion, radiation treatment significantly leads to weight loss, cardiac dysfunction and myocardial fibrosis, which can be ameliorated by consumption of DBT, involving regulation of Nrf2/HMGB1 pathway as probable underlying mechanisms.

## Data Availability

The original contributions presented in the study are included in the article/supplementary material, further inquiries can be directed to the corresponding authors.
